# Resident Medical Officers and Ward Sisters

**Published:** 1913-07-12

**Authors:** 


### RESIDENT MEDICAL OFFICERS AND WARD SISTERS.

### Friction the Exception; One Such Case.

One of the astonishing things about the working of our voluntary hospitals, year in year out, is the rarity of any appreciable friction between ward sisters and resident medical officers. Differences of opinion, even temporary coolnesses, are common enough, no doubt; but it is very seldom that sufficient animosity is aroused to prejudice the interests of the hospital and of its patients. Yet a situation apparently more calculated to provoke such friction it might be hard to devise or imagine; and it speaks volumes for the tact of both parties that collisions are so infrequent. Consider for a moment the relative positions of ward sister and resident medical officer. The former often middle-aged, never actually very young; experienced; accustomed to exercise authority over staff nurse, probationers, and ward maids; more or less permanent in her position. The latter nearly always very young; inexperienced; full of zeal and scientific enthusiasms; a bird of passage, institutionally; unaccustomed to authority, and exercising it perhaps for the first time; and technically the superior officer of the ward sister. Given an absence of tact and consideration on both sides, or even a temporary fit of irritability due to extraneous causes—a headache, or what not—and the train of combustion is so well and truly laid that an explosion may occur at any minute. Theoretically the system is impossible; actually, like so many of our slipshod British makeshift arrangements, it works admirably, save in very exceptional and occasional cases.

Such an unusual case has arisen recently in an English hospital, to judge by certain communications which have been submitted to us. Permission to name the parties and the institution concerned is not given; so our references to the matter are necessarily guarded. But since our advice has been solicited, it may be well to give a bare outline of the case, or rather of that side of it which has been presented to us. While a hospital resident medical officer was making his morning round in one of his wards he noticed a probationer dusting a bed. When outside the ward he asked the sister to instruct her probationers not to dust while he was going round. This the sister refused point blank to do; and, accusing the resident of interference with her affairs, added also an extremely uncomplimentary epithet. The resident informed her that he intended to complain of the whole incident to the Committee, which he did.

At this point we may remark that it surely would have been more courteous, and more in accordance with hospital procedure, if the resident had reported the circumstances to the matron before taking them to the Committee. It is a good plan not to refer to a committee any small matter which can with propriety be settled among the administrative officers themselves.

The Committee, after receiving the complaint, interviewed first the resident medical officer and then the sister, in each case without the presence of the other party. The sister denied using the uncomplimentary word as an epithet, though she admitted using a phrase in which it occurred, but not in application to the resident. The Committee then sent for another sister, and asked whether she had any cause of complaint against the resident medical officer: she mentioned two or three somewhat trivial derelictions of duty on the part of the latter.

The matter, we are informed, is closed; but one at least of the parties to the dispute is left with a rankling sense of injustice. It is contended, and we entirely agree, that the procedure of the Committee was wrong in two important respects. Both the resident and the sister should have appeared together before the Committee; so that the one could hear the complaint made against her, and the other the reply made to it. Secondly, to summon a third person (the other sister, that is) to make a statement as to past incidents not connected with the matter under consideration was injudicious, at the least; and to receive this statement in the absence of the resident medical officer, and without letting him know its nature, was absolutely unfair. It is injustice of this sort that undermines the basis of mutual accommodation and consideration without which the administration of a hospital cannot run smoothly.

Having said this much, we also feel bound to point out that the disputants have failed in some degree to exercise that tact and forbearance to which we alluded above. Without prejudging the case on such slender evidence as we possess, it is impossible to avoid the impression that part of the blame for this rests with the resident medical officer, whose dignity would seem to be as easily ruffled as his temper. Hospital sisters are not always the easiest of people to get on with; but no finer apprenticeship exists for the development in a resident medical officer of that tact and finesse, which are so valuable in medical practice, than in learning to conciliate, to manage, and to control a crotchety member of this much-tried and valuable class of nurse. From an experienced sister a resident can learn, if he goes the right way about it, an infinity of hints that will smooth his path in after years should he embark on "general practice." We cannot help suspecting that in the case now under discussion a little less self-importance, a little more good humour and *savoir faire*, would have saved all necessity for complaints and unpleasantness of every kind. We may be wrong, but our impression is that observance of the principles laid down in our opening paragraphs would have lubricated the relations of these hospital officers to such an extent that no friction would have occurred, or none sufficient to cause any appreciable degree of heat.

